# Protocatechuic acid promotes lactate synthesis in Sertoli cells of Tibetan sheep through AMPK/mTOR-mediated autophagy

**DOI:** 10.5713/ab.250776

**Published:** 2026-02-06

**Authors:** Xingcai Qi, Yi Wu, Qiao Li, Huihui Wang, Youji Ma

**Affiliations:** 1 College of Animal Science and Technology, Gansu Agricultural University, Lanzhou, China; 2 Gansu Key Laboratory of Animal Generational Physiology and Reproductive Regulation, Lanzhou, China

**Keywords:** AMP-activated Protein Kinase/Mechanistic Target of Rapamycin, Autophagy, Lactate, Protocatechuic Acid, Pyruvate

## Abstract

**Objective:**

This study aimed to investigate the effects of protocatechuic acid (PCA) on Sertoli cells (SCs) in Tibetan sheep, focusing on its impact on cell proliferation, lactate synthesis, and the underlying molecular mechanisms.

**Methods:**

Primary SCs were isolated from the testes of Tibetan sheep and subsequently cultured. The effects of PCA on the functionality of SCs were evaluated through *in vitro* experiments, with an additional investigation into its molecular mechanisms. *In vivo* and *in vitro* mouse models were employed to validate the regulatory mechanisms of PCA on male reproductive function and SCs.

**Results:**

PCA significantly upregulated the expression of proliferation and lactate synthesis-related genes in Tibetan sheep SCs, and increased the intracellular levels of pyruvate and lactate. Mechanistically, PCA activated the AMP-activated protein kinase (AMPK) pathway and inhibited the mechanistic target of rapamycin (mTOR) pathway, leading to the induction of autophagy in SCs. The use of autophagy inhibitors and AMPK inhibitors effectively blocked PCA-induced promotion of SCs proliferation and lactate synthesis. In mouse model studies, PCA demonstrated enhancements in sperm count, pregnancy rates, and litter size, while reduction in the rate of sperm abnormality. It also elevated activin A levels and decreased inhibin B levels in testicular tissues, increased serum follicle-stimulating hormone, luteinizing hormone, and testosterone levels, enhanced testicular antioxidant enzyme activities, and reduced malondialdehyde content. Furthermore, PCA upregulated the expression of proteins associated with proliferation and lactate synthesis in mouse testicular tissues and primary SCs, modulated the AMPK/mTOR pathway, and increased autophagic activity.

**Conclusion:**

PCA promotes proliferation and lactate synthesis in SCs of Tibetan sheep through AMPK/mTOR-mediated autophagy.

## INTRODUCTION

Tibetan sheep are a unique plateau-adapted breed indigenous to the Qinghai-Tibet Plateau, primarily distributed in high-altitude regions at 3,000–4,500 m above sea level. Compared to low-altitude sheep breeds, Tibetan sheep exhibit distinctive physiological characteristics such as delayed sexual maturity, lower fecundity, and longer growth and development cycles [1]. Moreover, Tibetan sheep have evolved unique adaptive mechanisms for energy metabolism, enhanced antioxidant capacity, and reproductive regulation [2–4]. These characteristics make Tibetan sheep an ideal model for studying reproductive processes and metabolic regulation, offering valuable insights into the mechanisms of male reproductive function in high-altitude environments.

Sertoli cells (SCs) are crucial somatic cells within the testes, providing essential nutrition and structural support for the development of germ cells at all stages, and serving as a fundamental prerequisite and guarantee for the production of healthy sperm [5]. Lactate produced through glycolysis in SCs serves as the primary energy source for germ cells, and alterations in its levels can significantly impact germ cell development, thereby affecting male fertility [6]. Studies have shown that lactate deficiency markedly reduces the vitality of male germ cells and induces apoptosis of spermatogenic cells [7]. For instance, specific knockout of the lactate dehydrogenase A (*LDHA*) gene in mice led to a significant decline in lactate content within SCs, resulting in reduced testicular weight and fertility [8]. Additionally, the infusion of lactate into the testes of adult cryptorchid rats effectively inhibited the loss of spermatocytes and sperm cells, significantly improving spermatogenesis [9]. These findings collectively highlight the crucial role of lactate metabolism in SCs for male reproductive function.

Research on SCs has achieved substantial progress in other sheep breeds. For instance, exposure of pregnant ewes to sewage sludge significantly reduced testicular weight and the number of SCs in male fetuses [10]. Combined exposure to molybdenum and cadmium decreased blood-testis barrier (BTB)-related protein levels in sheep SCs, thereby activating the reactive oxygen species (ROS)/Nucleotide-binding oligomerization domain (NOD)-like receptor pyrin domain-containing 3 (NLRP3) inflammasome pathway in testes to enhance ROS generation and inflammatory responses, ultimately inducing reproductive toxicity [11]. Additionally, dietary supplementation with linseed and grape seed tannin extracts facilitated seminiferous tubule development and increased the number of SCs in Hu lambs [12,13]. However, research on testicular SCs in Tibetan sheep is still limited, with a notable absence of systematic studies on their functional characteristics and metabolic adaptations under nutritional interventions or external stressors. This gap hinders a deeper understanding of male reproductive regulation in high-altitude environments.

Protocatechuic acid (PCA), also known as 3,4-dihydroxybenzoic acid, is a natural phenolic acid widely present in numerous vegetables and fruits. It is also a potent active ingredient in many traditional Chinese medicines, exhibiting a broad spectrum of pharmacological activities [14,15]. In terms of male reproduction, PCA has been shown to increase body weight in rats, enhance antioxidant enzyme activity and glutathione levels in the hypothalamus, testes, and epididymides, and significantly reduce biomarkers of inflammation and oxidative stress. Additionally, PCA significantly elevates serum levels of follicle-stimulating hormone (FSH), luteinizing hormone (LH), and testosterone (T), thereby positively influencing the reproductive function of male rats [16]. Furthermore, dietary supplementation with PCA effectively mitigates age-related declines in semen volume, sperm concentration, and total sperm count in breeding boars, while significantly improving sperm motility [17]. These studies collectively highlight the significant improvement effects of PCA on animal reproductive capacity.

However, research on the effect of PCA on testicular SCs function and its underlying molecular mechanisms remains limited, particularly in high-altitude-adapted species like Tibetan sheep. Given the critical role of SCs in supporting germ cell development and lactate synthesis, and the potential of PCA to improve reproductive function, this study aimed to investigate the impact of PCA on SCs proliferation and lactate synthesis and clarify the corresponding molecular mechanisms. We established an *in vitro* model of Tibetan sheep testicular SCs, which was complemented by validation in mouse models. The findings are expected to provide a theoretical basis for breeding high-quality rams and provide new insights into the application of PCA in improving male reproductive performance.

## MATERIALS AND METHODS

### Experimental animals and sample collection

In this study, three prepubertal (3 months old, 15±0.5 kg) male Tibetan sheep were purchased from the Ganjia Tibetan Sheep Breeding Cooperative in Xiahe County, Gansu Province, and forty prepubertal (3 weeks old, 15±0.5 g) male BALB/C mice were obtained from the Lanzhou Veterinary Research Institute, Chinese Academy of Agricultural Sciences.

For the Tibetan sheep experiment, testicular tissues were collected following euthanasia, and primary SCs were isolated and purified using a previously established laboratory protocol [18]. The key steps were as follows: Testicular tissues were surface-sterilized with 75% ethanol, decapsulated, and digested with trypsin and collagenase. Digestion was stopped by adding high-glucose Dulbecco’s Modified Eagle Medium/Nutrient Mixture F-12 (DMEM/F-12; Gibco, Cat. No. 11320033) supplemented with 12% fetal bovine serum (FBS), and the resulting cell suspension was filtered. Cells were then cultured in DMEM/F-12 (Gibco) supplemented with 12% FBS and 1% penicillin-streptomycin. After adhesion and proliferation, SCs were further purified using discontinuous Percoll density gradient centrifugation and repeated differential adhesion.

In the mice experiment, the animals were housed in plastic cages in a well-ventilated animal facility under a 12 h light/dark cycle. Before the experiment, the mice underwent a one-week acclimatization period with *ad libitum* access to food and water. Their mental state, diet, and excretion were monitored daily to ensure optimal health. The mice were randomly divided into four groups (n = 10 per group): a control group (normal feed+drinking water) and three experimental groups (normal feed+drinking water+different doses of PCA). The experimental groups received daily oral gavage of PCA at doses of 50 mg/kg, 100 mg/kg, and 200 mg/kg, respectively. During the experiment, body weight was measured weekly, and PCA doses were adjusted accordingly to maintain dosing accuracy. Daily observations of appearance characteristics and behavioral changes were recorded. The experimental period lasted 8 weeks. At the end of the experiment, the final body weight of the mice was recorded, and they were euthanized by cervical dislocation under mild ether anesthesia. Blood samples were collected from the posterior orbital venous plexus. Subsequently, testicular and epididymal tissues were excised, weighed, and processed for subsequent analyses. The epididymis was dissected to isolate sperm for the detection of relevant indicators. The left testicular tissues were partially cryopreserved at −80°C and partially fixed in 4% paraformaldehyde, while the right testicular tissues were used for primary SCs isolation following the procedure previously described in this study for Tibetan sheep SCs.

### Cell culture and identification

Primary SCs were isolated from the testes of Tibetan sheep and mice, respectively, and cultured in DMEM/F-12 with 12% FBS and 1% penicillin-streptomycin at 37°C in a 5% CO_2_ cell incubator, when cells reached approximately 80% confluence, they were used for subsequent experiments. The purity of the primary SCs was verified through immunofluorescence staining using only the specific marker for SCs: GATA binding protein 4 (GATA4). Antibody details are provided in Supplement 1.

### Immunofluorescence

Cells were fixed with 4% paraformaldehyde (Servicebio) for 30 min, permeated with 0.1% Triton X-100 (Servicebio) for 20 min, and blocked with 5% bovine serum albumin (BSA) for 30 min. Subsequently, the cells were incubated at 4°C in a humidified chamber with the primary antibody GATA4 (1:100 dilution; Bioss). Following primary antibody incubation, the cells were treated with a FITC-conjugated secondary antibody (1:200 dilution; Servicebio) and incubated in the dark at 37°C for 60 min. Nuclei were counterstained with 4′,6-diamidino-2-phenylindole (DAPI) staining solution and incubated in the dark at 37°C for 10 min. Finally, the samples were sealed with an anti-fade mounting medium and visualized under a fluorescence microscope (Apexbio).

### Cell viability assay

SCs were treated with varying concentrations of PCA (0, 40, 60, 100, 200, 400, 600, and 800 μM) for 48 h. Cell viability was then assessed using the cell counting kit-8 (CCK-8) assay (Biosharp) according to the manufacturer’s instructions.

### Quantitative real-time polymerase chain reaction

Total ribonucleic acid (RNA) was extracted using Trizol reagent (Solarbio) following the manufacturer’s instructions. Subsequently, complementary DNA (cDNA) was synthesized using the Evo M-MLV Plus cDNA Synthesis Kit (Accurate Biology) according to the manufacturer’s protocol. Gene expression levels were detected by quantitative real-time polymerase chain reaction (qPCR). The primer sequences used in this study are listed in Supplement 2. The relative expression levels of messenger RNA (mRNA) were analyzed using the comparative method (2^−^^ΔΔ^^Ct^) [19] to evaluate changes in gene expression.

### Cell culture duration optimization

To determine the optimal *in vitro* culture duration for Tibetan sheep SCs, a preliminary qPCR assay was performed to detect the mRNA expression levels of lactate synthesis-related key genes LDHA and glucose transporter 3 (GLUT3) in SCs at 24 h, 48 h, and 72 h. Based on the expression patterns of LDHA and GLUT3, the optimal duration was confirmed.

### Cell processing

PCA was dissolved in the culture medium, and cells were treated with different concentrations of PCA (40, 60, and 100 μM) for 48 h. After treatment, cell samples were collected for subsequent experiments.

To investigate the role of the autophagy signaling pathway, the autophagy inhibitor 3-methyladenine (3MA) was employed. A 5 mM solution of 3MA was prepared in DMEM/F-12 medium. Cells were pretreated with 5 mM 3MA for 1 h, followed by treatment with 100 μM PCA for 48 h. After treatment, cell samples were collected for further analysis.

To examine the role of the AMP-activated protein kinase (AMPK) signaling pathway, the AMPK inhibitor Compound C (CC) was used. A 10 μM solution of CC was prepared in DMEM/F-12 medium. Cells were pretreated with CC for 1 hour, followed by treatment with 100 μM PCA for 48 h. Subsequently, cell samples were collected for subsequent experiments.

### Western blot analysis

Total protein was extracted from cells and mice testicular tissues using radioimmunoprecipitation assay (RIPA) buffer supplemented with phenylmethylsulfonyl fluoride (PMSF) (Solarbio). The protein concentration was quantified using a bicinchoninic acid (BCA) protein assay kit (Beyotime). Western blot analysis was performed following a previously established protocol in the laboratory [18]. The antibodies used in this study are listed in Supplement 1. Finally, ImageJ software was employed to quantitatively analyze the Western blot results and evaluate the expression levels of the target proteins.

### Flow cytometry

Flow cytometry was employed to analyze apoptotic cells. Necrotic and apoptotic cells were detected by performing Annexin V-FITC (V-FITC)/Propidium Iodide (PI) double-labeling. An appropriate density of SCs was used to inoculate the complete culture medium contained in the wells of six-well plates. On reaching 70%–80% confluence, the cells were rinsed three times with PBS, and the SCs were digested and centrifuged at 100 ×g for 5 min. For necrosis and apoptosis analysis, the resulting cell pellets were resuspended in Annexin V labeled with fluorescent dye and PI staining solution, incubated at room temperature in the dark for 20 min, and then analyzed by flow cytometry.

### Transmission electron microscope analysis

Cell particles and testicular tissues were collected and subjected to double-layer fixation, dehydration, infiltration, embedding, ultrathin sectioning, and staining. Finally, the morphological structure of autophagosomes was observed and photographed using a transmission electron microscope (Hitachi).

### Measurement of lactate and pyruvate

#### For Tibetan sheep testicular primary Sertoli cells

An appropriate number of primary SCs were inoculated in a six-well plate and incubated for 48 h under different experimental conditions. After incubation and treatment, the culture supernatant was collected and the lactate content was determined according to the instructions provided with the lactate assay kit (Nanjing Jiancheng Bioengineering Institute). Simultaneously, the treated cells were harvested, and total proteins were extracted via ultrasonic disruption. The protein concentration was quantified using a BCA kit (Beyotime), and pyruvate content was determined following the instructions of the pyruvate assay kit (Nanjing Jiancheng Bioengineering Institute).

#### For mice testicular primary Sertoli cells

Primary SCs were isolated from mice pretreated with PCA *in vivo* and seeded into cell culture dishes. When cell confluence reached approximately 80% (approximately 7 d), both cells and supernatants were collected, and pyruvate and lactate levels were determined following the instructions of the same lactate and pyruvate assay kits as described above.

#### For mice testicular tissues

Testicular tissues under different treatment conditions were retrieved from a −80°C refrigerator and immediately homogenized using a pre-chilled homogenizer. Following centrifugation to remove tissues debris, the supernatant was collected for the determination of pyruvate and lactate levels in accordance with the instructions of the same lactate and pyruvate assay kits as described above.

### Semen quality detection

Sperm count: The cauda epididymis was ground thoroughly in normal saline, and the resulting sperm suspension was filtered through a nylon mesh to obtain purified sperm. A 5 μL filtrate was mixed with 95 μL formalin diluent containing sodium bicarbonate and trypan blue. Sperm counting was performed using a modified Neubauer hemocytometer under an optical microscope to assess sperm count.

#### Sperm morphology

A suitable amount of sperm suspension was added to a glass slide to prepare a smear, which was air-dried naturally. The smear was stained with hematoxylin and eosin (H&E) solution, followed by gentle rinsing with distilled water and air-drying. Observations were performed under an optical microscope at ×400 magnification, and at least 400 spermatozoa were morphologically assessed per sample, and the proportions of sperm with head, neck, tail, and total abnormalities were recorded, with results expressed as percentages.

### Mating test

Two weeks before the end of the 8-week PCA administration period, each male mouse was housed with two female mice with proven fertility (male-to-female ratio of 1:2). Female mice were checked daily in the early morning, and the presence of a vaginal plug served as a marker for successful mating. After separating male and female mice, the female mice were continuously monitored for 21 days. The pregnancy status of each female and the litter size per pregnant mouse were recorded, and the pregnancy rate and average litter size were calculated to assess the *in vivo* fertility of the male mice.

### Measurement of reproductive hormones

After collecting blood samples from the mice, the serum was separated by centrifugation (1,000 ×g, 10 min, 4°C). Following the operating instructions of the FSH, LH, and T detection kits (Inova), the concentrations of FSH, LH, and T in the serum were determined using enzyme-linked immunosorbent assay (ELISA).

### Measurement of oxidative stress index and activin A/inhibin B

Testicular tissues were rinsed with pre-cooled normal saline to remove blood and impurities. After weighing, it was homogenized in pre-cooled phosphate buffer (PBS, 0.1 M, pH 7.4) at a 1:9 (w/v) ratio under ice bath conditions. The homogenate was centrifuged (5,000 ×g, 15 min, 4°C), and the supernatant was collected. Following the kit instructions, the levels of superoxide dismutase (SOD), glutathione peroxidase (GSH-Px), malondialdehyde (MDA), total antioxidant capacity (T-AOC), and catalase (CAT) (Nanjing Jiancheng Bioengineering Institute Kit) were measured to evaluate oxidative stress-related indicators. Additionally, concentrations of activin A and inhibin B in testicular tissues were quantified using specific ELISA kits (Shanghai Meiyan Biotechnology).

### Histological staining

The testicular tissues were fixed in 4% paraformaldehyde, dehydrated, embedded in paraffin, and sectioned into 5 μm thick slices. The sections were then stained with H&E and examined under an optical microscope (Olympus).

### Statistical analysis

Data were visualized using GraphPad Prism 9.0 software. Statistical analysis was performed using SPSS ver. 25.0, employing one-way analysis of variance and significance tests. Results are expressed as mean±standard deviation (mean±SD), p< 0.05 was considered statistically significant.

## RESULTS

### Identification of Tibetan sheep primary Sertoli cells and the effect of protocatechuic acid on Sertoli cells viability

GATA4 protein was positively expressed in the isolated primary SCs (Supplement 3A), confirming the successful isolation of Tibetan sheep primary SCs. After 48 h of PCA treatment (0, 40, 60, 100, 200, 400, 600, 800 μM), SCs viability showed no significant difference at 0–100 μM, was significantly increased at 200 μM, and markedly reduced at 600 μM (Supplements 3B, 3C). Based on these findings, PCA concentrations of 0, 40, 60, and 100 μM were selected for subsequent experiments.

The qPCR results revealed that the mRNA expression levels of *LDHA* and *GLUT3* were significantly higher at 48 h compared with 24 h and 72 h, indicating that the lactate synthesis capacity of SCs was the strongest at this time point. Therefore, 48 h was selected as the optimal *in vitro* culture duration for SCs (Supplement 3D).

### Protocatechuic acid promotes Tibetan sheep primary Sertoli cells proliferation and lactate synthesis

**Figure 1:**
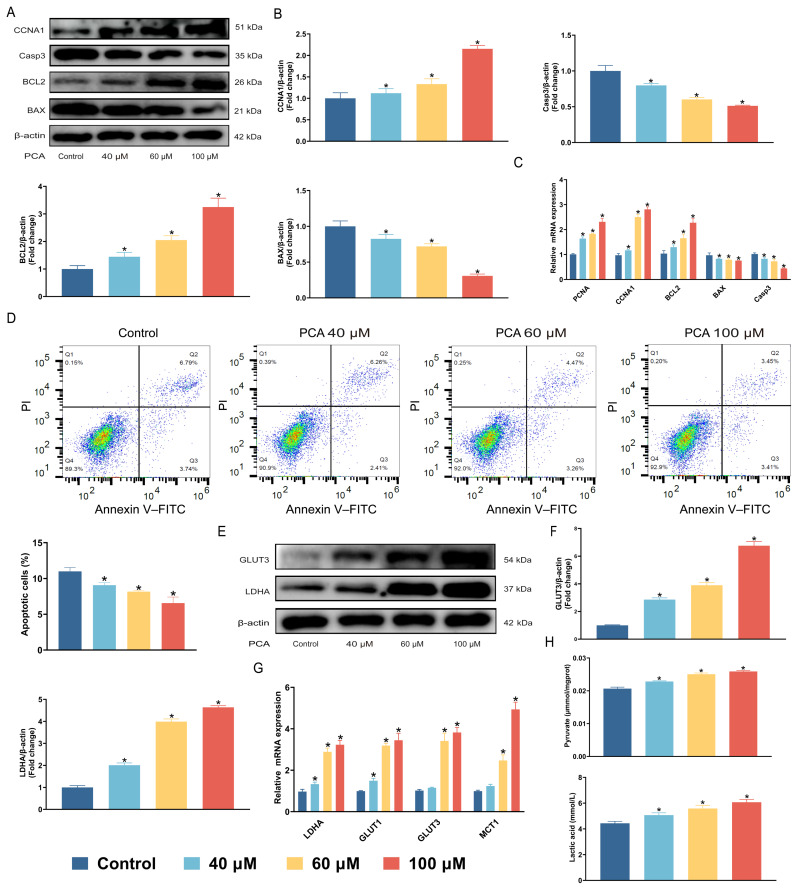
PCA promotes proliferation and lactate synthesis in Tibetan sheep primary SCs. (A) Protein levels of CCNA1, Casp3, BCL2, and BAX detected by Western blot. (B) Quanti-tative analysis of protein bands in panel A. (C) mRNA levels of *PCNA*, *CCNA1*, *Casp3*, *BCL2*, and *BAX* detected by qPCR. (D) Apoptosis level of cells was detected by flow cytometry. (E) Protein levels of GLUT3 and LDHA detected by Western blot. (F) Quantitative analysis of protein bands in panel E. (G) mRNA expression of *LDHA*, *GLUT1*, *GLUT3*, and *MCT1* detected by qPCR. (H) Levels of pyruvate and lactate in SCs were determined by commercial assay kits. Data are presented as the mean±SD. * p<0.05 vs. control group. PCA, protocatechuic acid; SCs, Sertoli cells; CCNA1, cyclin A1; Casp3, caspase 3; BCL2, B-cell lymphoma 2; BAX, BCL2-associated X protein; mRNA, messenger RNA; PCNA, proliferating cell nuclear antigen; qPCR, quantitative real-time polymerase chain reaction; GLUT3, glucose transporter 3; LDHA, lactate dehydrogenase A; GLUT1, glucose transporter 1; MCT1, monocarboxylate transporter 1; SD, standard deviation.

Compared with the control group, the protein expression levels of cyclin A1 (CCNA1) and B-cell lymphoma 2 (BCL2) significantly increased with the PCA concentration. Similarly, the mRNA expression levels of proliferating cell nuclear antigen (*PCNA*), *CCNA1*, and *BCL2* were also markedly elevated. Conversely, the mRNA and protein expression levels of apoptosis-related genes caspase 3 (Casp3) and BCL2-associated X protein (BAX) were significantly reduced (Figures 1A–1C). To further investigate the anti-apoptotic effect of PCA, flow cytometric analysis was performed using Annexin V-FITC/PI staining. Cells were classified into four distinct populations: viable cells (Annexin V−/PI−), early apoptotic cells (Annexin V+/PI−), late apoptotic or necrotic cells (Annexin V+/PI+), and mechanically damaged cells (Annexin V−/PI+). The total apoptotic cell population, calculated as the sum of early and late apoptotic cells, was significantly reduced in PCA-treated groups compared to the control (Figure 1D).

In addition, PCA treatment at the same time and concentration range significantly upregulated the protein expression levels of GLUT3 and LDHA (Figures 1E, 1F). The mRNA expression levels of lactate synthesis-related genes *LDHA*, glucose transporter 1 (*GLUT1*), *GLUT3*, and monocarboxylate transporter 1 (*MCT1*) were significantly increased in SCs (Figure 1G). Furthermore, the intracellular levels of pyruvate and lactate in SCs were significantly elevated (Figure 1H).

### Protocatechuic acid promotes Tibetan sheep primary Sertoli cells proliferation and lactate synthesis by activating autophagy

Compared with the control group, the expression of autophagy marker proteins beclin 1 (BECN1) and microtubule-associated protein 1 light chain 3 beta-II (LC3II), as well as the LC3II/LC3I ratio, increased significantly with the increase of PCA concentration, while the expression of sequestosome 1 (SQSTM1) significantly decreased (Supplements 4A, 4B). Electron microscopy further confirmed that, compared with the control group, the number of autophagolysosomes increased significantly with higher PCA concentrations (Supplements 4C, 4D).

**Figure 2:**
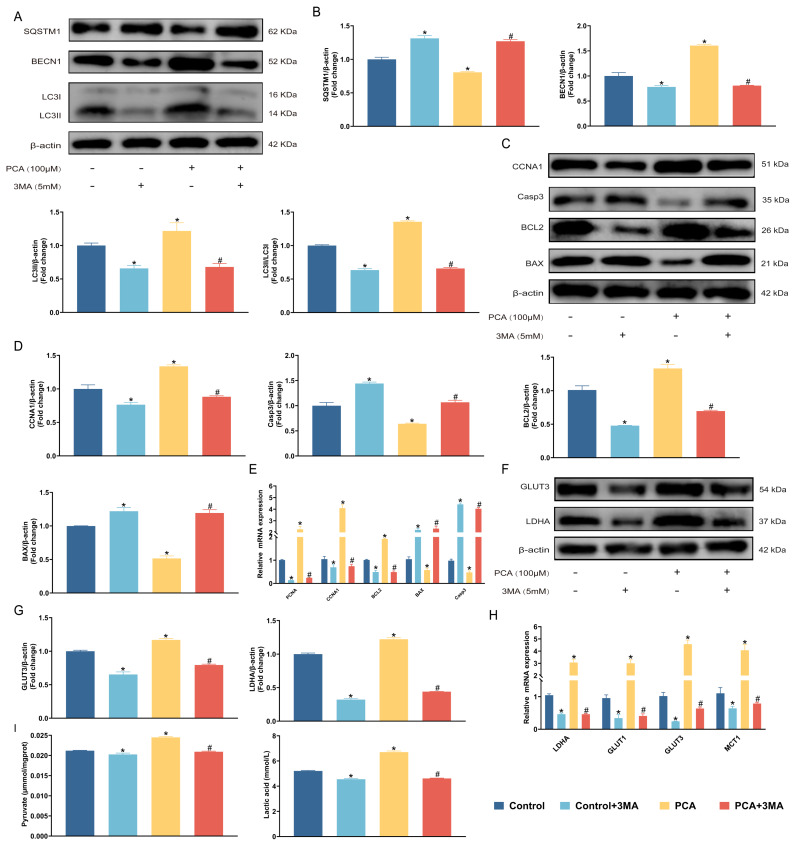
PCA promotes proliferation and lactate synthesis in primary Tibetan sheep SCs by activating autophagy. (A) Protein levels of SQSTM1, BECN1, and LC3II detected by Western blot. (B) Quantitative analysis of protein bands in panel A. (C) Protein levels of CCNA1, Casp3, BCL2, and BAX detected by Western blot. (D) Quantitative analysis of protein bands in panel C. (E) mRNA levels of *PCNA*, *CCNA1*, *Casp3*, *BCL2*, and *BAX* detected by qPCR. (F) Protein levels of GLUT3 and LDHA detected by Western blot. (G) Quantitative analysis of pro-tein bands in panel F. (H) mRNA expression of *LDHA*, *GLUT1*, *GLUT3*, and *MCT1* detected by qPCR. (I) Levels of pyruvate and lactate in SCs were determined by commercial assay kits. Data are presented as the mean±SD. * p<0.05 vs. control group. # p<0.05 vs. PCA group. PCA, protocatechuic acid; SCs, Sertoli cells; SQSTM1, sequestosome 1; BECN1, beclin 1; LC3II, microtubule-associated protein 1 light chain 3 beta-II; CCNA1, cyclin A1; Casp3, caspase 3; BCL2, B-cell lymphoma 2; BAX, BCL2-associated X protein; mRNA, messenger RNA; PCNA, proliferating cell nuclear antigen; qPCR, quantitative real-time polymerase chain reaction; GLUT3, glucose transporter 3; LDHA, lactate dehydrogenase A; GLUT1, glucose transporter 1; MCT1, monocarboxylate transporter 1; SD, standard deviation.

Pretreatment with 5 mM 3MA, compared with the PCA group, BECN1, LC3II levels and LC3II/LC3I ratio were decreased, SQSTM1 levels were increased (Figures 2A, 2B). The protein expression levels of CCNA1 and BCL2 were markedly reduced. Similarly, the mRNA expression of cell proliferation-related genes *PCNA*, *CCNA1*, and *BCL2* was also significantly decreased. Conversely, both the mRNA and protein expression levels of apoptosis-related genes Casp3 and BAX were significantly increased (Figures 2C–2E). Furthermore, the protein levels of LDHA, GLUT3, along with the mRNA levels of *LDHA*, *GLUT1*, *GLUT3*, *MCT1* were downregulated (Figures 2F–2H). The intracellular levels of pyruvate and lactate in SCs were also significantly decreased (Figure 2I).

### Protocatechuic acid promotes Tibetan sheep primary Sertoli cells proliferation and lactate synthesis by regulating the AMP-activated protein kinase/mechanistic target of rapamycin pathway mediated autophagy

Compared with the control group, the protein expression of phosphorylated AMP-activated protein kinase (p-AMPK) and its ratio to AMPK significantly increased, while the expression of pathway-related phosphorylated mechanistic target of rapamycin (p-mTOR) protein and its ratio to mTOR significantly decreased with increasing PCA concentration (Supplements 5A, 5B).

**Figure 3:**
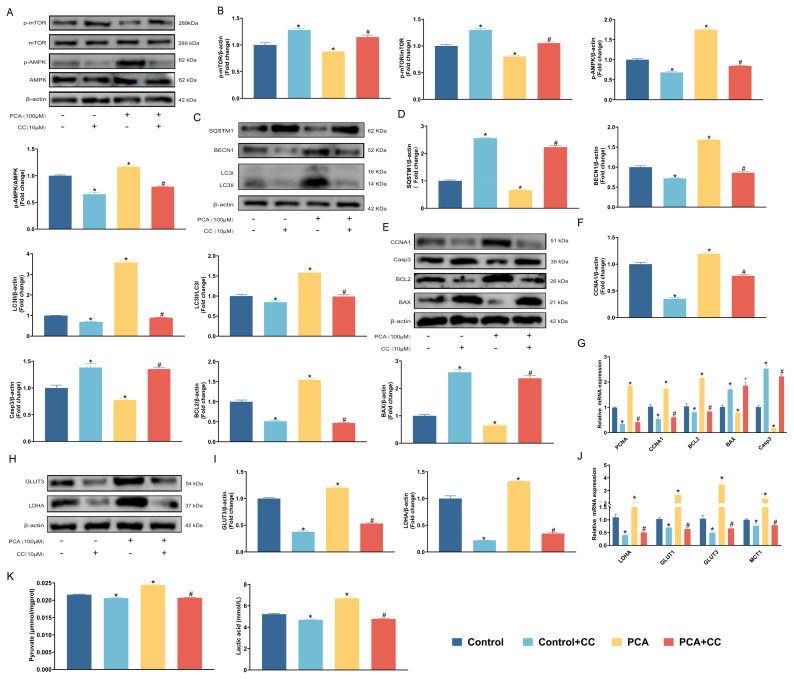
PCA promotes proliferation and lactate synthesis in primary Tibetan sheep SCs by regulating the AMPK/mTOR pathway-mediated autophagy. (A) Protein levels of p-mTOR, mTOR, p-AMPK, and AMPK detected by Western blot. (B) Quantitative analysis of protein bands in panel A. (C) Protein levels of SQSTM1, BECN1, and LC3II detected by Western blot. (D) Quantitative analysis of protein bands in panel C. (E) Protein levels CCNA1, Casp3, BCL2, and BAX detected by Western blot. (F) Quantitative analysis of protein bands in panel E. (G) mRNA levels of *PCNA*, *CCNA1*, *Casp3*, *BCL2*, and *BAX* detected by qPCR. (H) Protein levels of GLUT3 and LDHA detected by Western blot. (I) Quantitative analy-sis of protein bands in panel H. (J) mRNA expression of *LDHA*, *GLUT1*, *GLUT3*, and *MCT1* de-tected by qPCR. (K) Levels of pyruvate and lactate in SCs were determined by commercial assay kits. Data are presented as the mean±SD. * p<0.05 vs. control group. # p<0.05 vs. PCA group. PCA, protocatechuic acid; SCs, Sertoli cells; p-mTOR, phosphorylated mechanistic target of rapamycin; mTOR, mechanistic target of rapamycin; p-AMPK, phosphorylated AMP-activated protein kinase; AMPK, AMP-activated protein kinase; SQSTM1, sequestosome 1; BECN1, beclin 1; LC3II, light chain 3 beta-II; CCNA1, cyclin A1; Casp3, caspase 3; BCL2, B-cell lymphoma 2; BAX, BCL2-associated X protein; mRNA, messenger RNA; PCNA, proliferating cell nuclear antigen; qPCR, quantitative real-time polymerase chain reaction; GLUT3, glucose transporter 3; LDHA, lactate dehydrogenase A; GLUT1, glucose transporter 1; MCT1, monocarboxylate transporter 1; SD, standard deviation.

Pretreatment with 10 μM CC, compared with the PCA group, p-AMPK level and p-AMPK/AMPK ratio were reduced, p-mTOR level and p-mTOR/mTOR ratio were increased (Figures 3A, 3B); LC3II, BECN1 levels and LC3II/LC3I ratio were decreased, SQSTM1 level was increased (Figures 3C, 3D). Additionally, the protein expression levels of CCNA1 and BCL2 were markedly reduced. Similarly, the mRNA expression of cell proliferation-related genes *PCNA*, *CCNA1*, and *BCL2* was also significantly decreased. Conversely, both the mRNA and protein expression levels of apoptosis-related genes Casp3 and BAX were significantly increased (Figures 3E–3G). Furthermore, the protein levels of LDHA, GLUT3, along with the mRNA levels of *LDHA*, *GLUT1*, *GLUT3*, *MCT1* were downregulated (Figures 3H–3J). The intracellular levels of pyruvate and lactate in SCs were also significantly decreased (Figure 3K).

### Protocatechuic acid enhances reproductive function and testicular antioxidant capacity in mice

**Figure 4:**
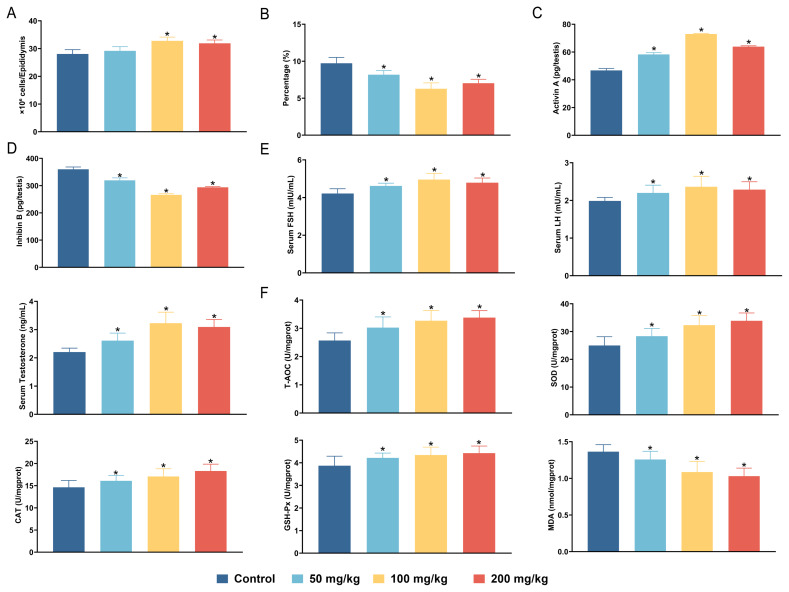
PCA enhances reproductive function and testicular antioxidant capacity in mice. (A) Epididymal sperm count in mice detected by hemocytometer counting. (B) Total sperm abnormality rate in mice detected by hematoxylin and eosin staining. (C) Concentra-tions of activin A in testicular tissues of mice detected by ELISA. (D) Concentrations of inhibin B in testic-ular tissues of mice detected by ELISA. (E) Concentrations of FSH, LH, and T in serum of mice detected by ELISA. (F) Activities of T-AOC, SOD, CAT, and GSH-Px, and MDA in testicular tissues of mice detected by ELISA. Data are presented as the mean±SD. * p<0.05 vs. control group. PCA, protocatechuic acid; ELISA, enzyme-linked immunosorbent assay; FSH, follicle-stimulating hormone; LH, luteinizing hormone; T, testosterone; T-AOC, total antioxidant capacity; SOD, superoxide dismutase; CAT, catalase; GSH-Px, glutathione peroxidase; MDA, malondialdehyde; SD, standard deviation.

After 8 weeks of treatment with PCA at doses of 50, 100, and 200 mg/kg, male mice showed a significant increase in epididymal sperm count and a marked decrease in sperm malformation rate (Figures 4A, 4B). Mating experiments revealed higher pregnancy rates of female mice mated with PCA-treated males and significantly elevated average litter sizes (Supplement 6). PCA treatment elevated activin A levels and decreased inhibin B levels in testicular tissues (Figures 4C, 4D), and increased serum concentrations of FSH, LH, and T (Figure 4E). Notably, the most pronounced changes across these reproductive parameters were observed in the 100 mg/kg group. Regarding testicular antioxidant capacity, the activities of T-AOC, SOD, CAT, and GSH-Px were significantly enhanced, whereas MDA levels were significantly reduced (Figure 4F). There were no significant differences in testicular weight among groups, and testicular structure remained intact (Supplements 7A, 7B).

### Protocatechuic acid enhances proliferation and lactate synthesis in testes of mice and primary Sertoli cells

**Figure 5:**
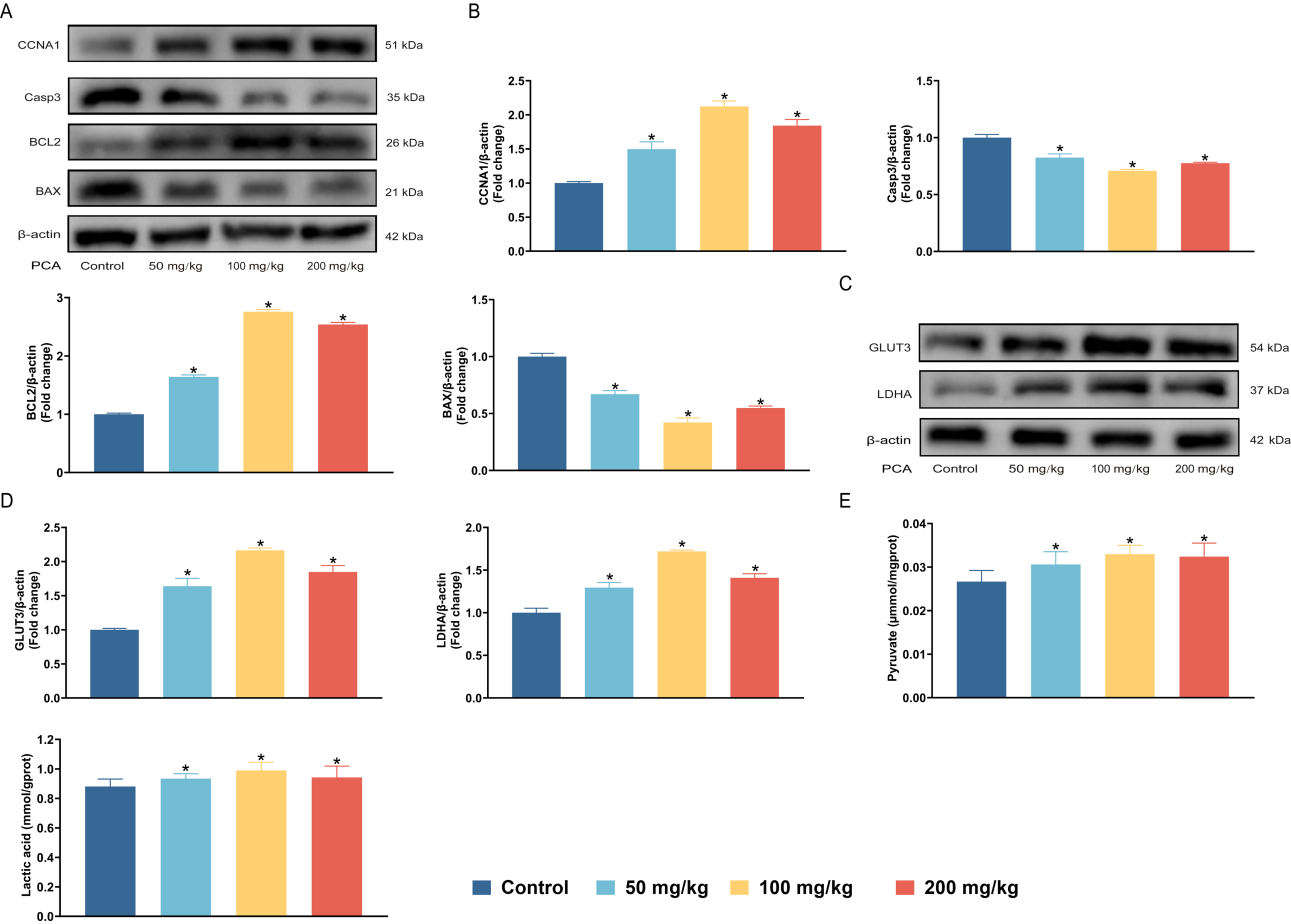
PCA enhances proliferation and lactate synthesis in testicular tissues of mice. (A) Protein levels of CCNA1, Casp3, BCL2, and BAX detected by Western blot. (B) Quantitative analysis of protein bands in panel A. (C) Protein levels of GLUT3 and LDHA detected by Western blot. (D) Quantitative analysis of protein bands in panel C. (E) Levels of pyruvate and lactate in testicular tissues of mice were detected by commercial assay kits. Data are presented as the mean±SD. * p<0.05 vs. control group. CCNA1, cyclin A1; Casp3, caspase 3; BCL2, B-cell lymphoma 2; BAX, BCL2-associated X protein; GLUT3, glucose transporter 3; LDHA, lactate dehydrogenase A; PCA, protocatechuic acid; SD, standard deviation.

GATA4 was positively expressed in the isolated primary SCs (Supplement 8A), confirming the successful isolation of primary SCs of mice. PCA treatment upregulated the protein expression levels of CCNA1 and BCL2 in both testicular tissues of mice and primary SCs. Conversely, the protein levels of apoptosis-related genes Casp3 and BAX were significantly decreased (Figures 5A, 5B; Supplements 8B, 8C).

Additionally, the protein expression levels of LDHA and GLUT3 were significantly increased in both testicular tissues of mice and primary SCs (Figures 5C, 5D; Supplements 8D, 8E). Furthermore, PCA treatment significantly increased pyruvate and lactate levels in both testicular tissues and primary SCs (Figure 5E; Supplement 8F).

### Protocatechuic acid regulates AMP-activated protein kinase/mechanistic target of rapamycin signaling pathway and induces autophagy in testes of mice and primary Sertoli cells

**Figure 6:**
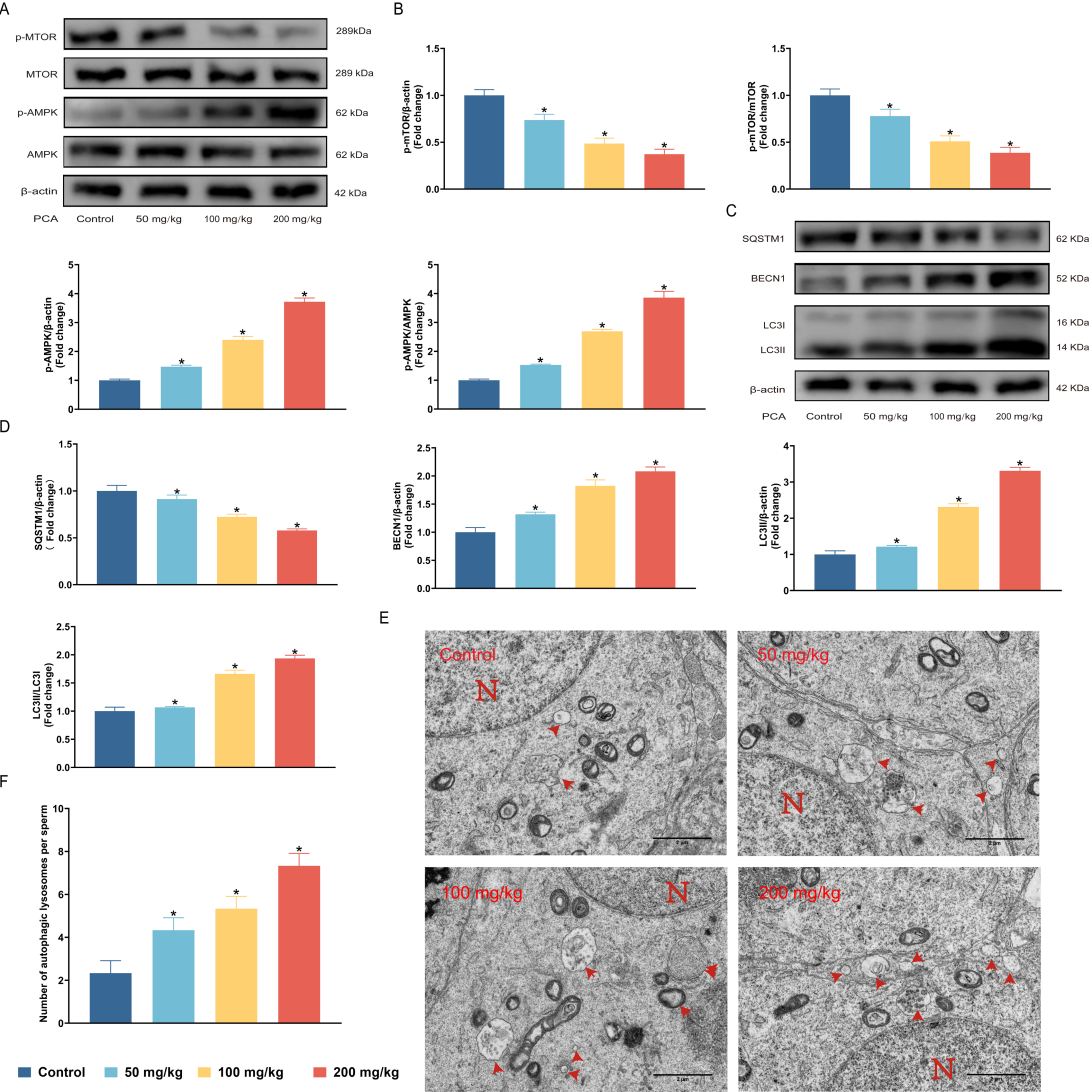
PCA regulates AMPK/mTOR signaling pathway and enhances autophagy in testicular tissues of mice. (A) Protein levels of p-mTOR, mTOR, p-AMPK, and AMPK detected by Western blot. (B) Quantitative analysis of protein bands in panel A. (C) Protein levels of SQSTM1, BECN1, and LC3II detected by Western blot. (D) Quantitative analysis of protein bands in panel C. (E) Autophagy levels were evaluated by transmission electron micrographs. The red arrows indicate autophagic lysosomes, and “N” denotes the nucleus. (F) Quantitative analysis of panel E. Data are presented as the mean±SD. * p<0.05 vs. control group. p-mTOR, phosphorylated mechanistic target of rapamycin; mTOR, mechanistic target of rapamycin; AMPK, AMP-activated protein kinase; PCA, protocatechuic acid; SQSTM1, sequestosome 1; BECN1, beclin 1; LC3II, light chain 3 beta-II; SD, standard deviation.

PCA treatment significantly increased the level of p-AMPK and the p-AMPK/AMPK ratio in testicular tissues of mice and primary SCs, while the levels of p-mTOR and the p-mTOR/mTOR ratio were significantly decreased (Figures 6A, 6B; Supplements 9A, 9B). Additionally, the protein levels of BECN1, LC3II, and LC3II/LC3I ratio were significantly increased, whereas the level of SQSTM1 was significantly decreased (Figures 6C, 6D; Supplements 9C, 9D). Furthermore, the number of autophagolysosomes was notably increased in the testes of the mice (Figures 6E, 6F).

## DISCUSSION

Our study demonstrates that PCA significantly enhances proliferation and lactate synthesis in Tibetan sheep testicular primary SCs by regulating the AMPK/mTOR pathway and inducing autophagy. Specifically, it upregulates the expression of proliferation- and lactate synthesis-related genes/proteins, increases intracellular pyruvate and lactate levels, and these effects were reversed by the autophagy inhibitor and AMPK inhibitor, confirming the core mediating role of the AMPK/mTOR pathway. *In vivo* experiments in mice further verified that PCA promotes the expression of proliferation- and lactate synthesis-related proteins, raises pyruvate and lactate contents, regulates the AMPK/mTOR pathway, enhances autophagic activity in testicular tissues and primary SCs of mice. Furthermore, PCA administration improved the antioxidant capacity of testicular tissues, increased epididymal sperm count, reduced sperm malformation, and enhanced pregnancy rates and litter size, accompanied by elevated serum levels of FSH, LH, and T. Collectively, these results indicate that PCA is a promising natural agent for improving male reproductive function and provide novel insights into SCs regulatory mechanisms.

PCA is the primary metabolite of complex polyphenols in vegetables, fruits, and herbs, and possesses diverse pharmacological properties [20]. Research has shown that PCA exerts beneficial effects on animal reproduction. For instance, PCA significantly reduces BPA-induced apoptosis and mitigates BPA related reproductive damage [21]. Additionally, as an active component of Cynomorium songaricum, PCA prevents the occurrence of benign prostatic hyperplasia in rats by inhibiting the expression of PCNA in BPH-1 cells induced by dihydrotestosterone or 17β-estradiol. Moreover, PCA reduces the expression of prostate androgen receptors, estrogen receptors α/β, and steroid 5-alpha reductase [22]. In our study, we demonstrated that PCA exerts consistent regulatory effects on both Tibetan sheep and mice testicular primary SCs, as well as mice testicular tissues. Specifically, PCA promoted SCs proliferation by upregulating the expression of CCNA1 and BCL2, while inhibiting the expression of pro-apoptotic genes Casp3 and BAX. Concomitantly, PCA significantly upregulated the expression of lactate synthesis-related key genes LDHA and GLUT3, and consequently increased lactate levels in SCs cultures and mouse testes. Collectively, these results clearly illustrate that PCA effectively enhances lactate synthesis, promotes proliferation, and suppresses apoptosis in primary SCs from Tibetan sheep and mice, thereby improving SCs physiological function.

In addition, accumulating evidence indicates that PCA exerts protective effects on male reproductive function under various pathological conditions, primarily through enhancing antioxidant capacity, suppressing inflammation, and restoring hormonal homeostasis [23,24]. In agreement with these observations, our *in vivo* data show that PCA increases epididymal sperm count, improves sperm quality, pregnancy rate, and average litter size in mice, accompanied by significantly increased activities of T-AOC, SOD, CAT, and GSH-Px in testicular tissues, along with reduced MDA levels, indicating an overall improvement in testicular redox homeostasis.

Given the central role of SCs in linking spermatogenesis with endocrine feedback regulation, we further examined whether PCA-induced improvements in SCs function were associated with alterations in the hypothalamic-pituitary-gonadal (HPG) axis. Activin A and inhibin B, both predominantly produced by SCs, are key regulators of pituitary gonadotropin secretion [25]. Acting antagonistically, inhibin B provides the principal negative feedback on FSH synthesis and release, whereas activin A enhances pituitary gonadotroph responsiveness to gonadotropin-releasing hormone via Smad2/3 signaling, thereby promoting LH secretion and Leydig cells T production [26,27]. At the molecular level, inhibin B antagonizes activin signaling through interactions with betaglycan and type II activin receptors, preventing excessive FSH stimulation and maintaining testicular homeostasis [28]. Although PCA has been reported to increase circulating FSH, LH, and T levels [16,24], the underlying regulatory mechanisms remain unclear. In this study, PCA increased testicular activin A while reducing inhibin B levels, concomitant with elevated serum FSH, LH, and T. This altered activin A/inhibin B balance likely attenuates negative feedback on pituitary FSH secretion and enhances gonadotropic axis activity. Nevertheless, given the multilevel regulation of the HPG axis, potential direct actions of PCA at the hypothalamic and pituitary levels cannot be excluded and warrant further investigation.

Autophagy is an intracellular catabolic process that participates in cell development and maintains homeostasis [29]. Studies have shown that autophagy participates in the development of mammalian testes and is closely related to spermatogenesis, hormone secretion, and the maintenance of testicular microenvironment stability. Excessive or insufficient autophagy can affect testicular function, normal spermatogenesis, and reproduction [30]. Studies have shown that destroying the normal autophagy level of mice SCs can lead to abnormal sperm structure and produce abnormal sperm [31]. During the aging process of mice, the level of autophagy in SCs is reduced, leading to the morphology of the testes gradually changing, its function being degraded, and the impairment of BTB function, affecting the reproductive function of mice [32]. Increasing the level of autophagy in mice SCs can attenuate the reproductive damage caused by oxidative stress and improve sperm motility [33]. Consistently, our experiments on primary Tibetan sheep testicular SCs demonstrated that PCA significantly enhances the autophagy level in SCs, after adding the autophagy inhibitor 3MA, the promoting effects of PCA on SCs proliferation and lactate synthesis were blocked. Furthermore, *in vivo* experiments confirmed that PCA significantly increases the autophagy level in mice testes and primary SCs. These findings suggest that PCA promotes the proliferation and lactate synthesis of primary SCs and mice testes by activating the autophagy pathway.

AMPK is an intracellular energy sensor that is involved in regulating cell metabolism and maintaining energy homeostasis [34]. Studies have shown that bta-miR-365-3p targets FK506-binding protein 5 (FKBP5) to modulate preadipocyte differentiation in Yanbian cattle via the AMPK/mTOR signaling pathway [35]. This example illustrates one of the various cellular processes regulated by the AMPK/mTOR pathway. In the context of animal reproduction, the AMPK/mTOR pathway induces autophagy by negatively regulating the mTOR signaling pathway, thereby modulating reproductive functions. For example, activation of the AMPK/mTOR signaling pathway in mouse spermatogonia and increased autophagy levels can effectively alleviate CuSO_4_-induced testicular injury and spermatogenesis impairment [36]. Melatonin promotes autophagy through its receptor-mediated AMPK/mTOR pathway, thereby enhancing progesterone secretion in sheep luteal cells [37]. BMSC-Exos promotes autophagy by activating the AMPK/mTOR signaling pathway, thereby alleviating the degradation of the BTB function of testicular SCs caused by aging [38]. Venlafaxine and carvedilol effectively improve testicular injury and spermatogenesis disorders in patients with rheumatoid arthritis by targeting the AMPK/ERK and PI3K/AKT/mTOR pathways [39]. Sitagliptin increases autophagy levels in rats by targeting the AMPK/mTOR pathway, thereby reducing cadmium-induced testicular injury [40]. These studies collectively indicate that the AMPK/mTOR pathway is a key regulatory node in reproductive function by mediating autophagy. In our study, experiments using Tibetan sheep primary testicular SCs demonstrated that PCA modulates the AMPK/mTOR signaling pathway. Notably, treatment with the AMPK inhibitor CC not only significantly decreased autophagic activity in SCs but also abrogated the PCA-induced promotion of SCs proliferation and lactate synthesis. Furthermore, *in vivo* experiments in mice confirmed that PCA effectively regulates the AMPK/mTOR signaling pathway in both testicular tissues and primary SCs. These results indicate that PCA promotes the proliferation and lactate synthesis of primary SCs and mice testes through AMPK/mTOR pathway mediated autophagy.

## CONCLUSION

**Figure 7:**
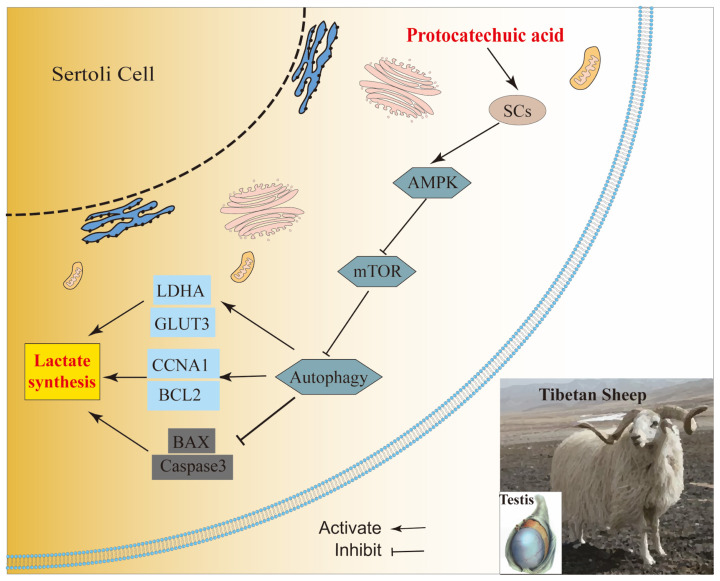
Graphical abstract. Schematic diagram illustrating the mechanism by which PCA promotes lactate synthesis in SCs of Tibetan sheep through AMPK/mTOR-mediated autophagy. SCs, Sertoli cells; AMPK, AMP-activated protein kinase; mTOR, mechanistic target of rapamycin; LDHA, lactate dehydrogenase A; GLUT3, glucose transporter 3; CCNA1, cyclin A1; BCL2, B-cell lymphoma 2; BAX, BCL2-associated X protein; PCA, protocatechuic acid.

In summary, PCA promotes lactate synthesis in SCs and mice testes by regulating the AMPK/mTOR pathway mediated autophagy (Figure 7). This study not only revealed the molecular mechanism of PCA in regulating male reproductive function but also provided an important theoretical basis for the cultivation of excellent Tibetan rams. Meanwhile, this research lays a solid scientific foundation for the potential application of PCA in livestock production and male reproductive health, demonstrating significant theoretical value and practical significance.

## Data Availability

Upon reasonable request, the datasets of this study can be available from the corresponding author.

## SUPPLEMENTARY MATERIAL

**Supplement 1.** Information of antibodies used in this study.

**Supplement 2.** Information of primers used in this study.

**Supplement 3.** Identification of Tibetan sheep primary Sertoli cells (SCs) and screening of concentration and treatment duration.

**Supplement 4.** PCA activates autophagy in Tibetan sheep primary SCs.

**Supplement 5.** PCA regulates AMP-activated protein kinase (AMPK)/mechanistic target of rapamycin (mTOR) pathway in Tibetan sheep primary SCs.

**Supplement 6.** Effects of PCA on reproductive performance of male mice.

**Supplement 7.** Effects of PCA on testicular weight and morphology in mice.

**Supplement 8.** PCA enhances proliferation and lactate synthesis in primary SCs of mice.

**Supplement 9.** PCA regulates AMPK/mTOR signaling pathway and enhances autophagy in primary SCs of mice.
