# Supplementary material for: Protocatechuic acid promotes lactate synthesis in Sertoli cells of Tibetan sheep through AMPK/mTOR-mediated autophagy
Source: Anim Biosci. 2026 Feb 6;39(6):250776. doi: 10.5713/ab.250776 (PMC13243928; doi:10.5713/ab.250776)
Supplement: Supplementary file 1 [file ab-250776-Supplementary-1.pdf]

Supplement 1. Information of antibodies used in this study

Name	Manufacturer	Cat. No.	Application	Dilution
GATA4	Bioss, Beijing, China	bs-1778R	Cell immunofluorescence	1:100
CCNA1	Bioss, Beijing, China	bs-5739R	Western Blot	1:500
CASP3	Bioss, Beijing, China	bs-0081R	Western Blot	1:500
BCL2	Bioss, Beijing, China	bsm-33411M	Western Blot	1:500
BAX	Bioss, Beijing, China	bsm-52316R	Western Blot	1:500
β-actin	Proteintech, Wuhan, China	81115-1-RR	Western Blot	1:5000
LDHA	Proteintech, Wuhan, China	21799-1-AP	Western Blot	1:5000
GLUT3	Proteintech, Wuhan, China	20403-1-AP	Western Blot	1:1000
LC3	Abmart, Shanghai, China	T55992	Western Blot	1:1000
SQSTM1	Proteintech, Wuhan, China	18420-1-AP	Western Blot	1:5000
BECN1	Proteintech, Wuhan, China	11306-1-AP	Western Blot	1:1000
mTOR	Abmart, Shanghai, China	T55306	Western Blot	1:1000
p-mTOR	Abmart, Shanghai, China	T56571	Western Blot	1:1000
AMPK	Affinity, USA	AF6423	Western Blot	1:1000
p-AMPK	Affinity, USA	AF3423	Western Blot	1:1000
