# Supplementary material for: Protocatechuic acid promotes lactate synthesis in Sertoli cells of Tibetan sheep through AMPK/mTOR-mediated autophagy
Source: Anim Biosci. 2026 Feb 6;39(6):250776. doi: 10.5713/ab.250776 (PMC13243928; doi:10.5713/ab.250776)
Supplement: Supplementary file 2 [file ab-250776-Supplementary-2.pdf]

Supplement 2. Information of primers used in this study

Gene	GenBank No.	Sequence (5′–3′)	Amplicon size (bp)	Application	Species
CCNA1	XM_042255137	F: CACAGTTTCCCCTATGCTGGT<br>R: GCCTTGGGCCTGTGTCTTAT	150bp	qRT-PCR	Sheep
PCNA	XM_004014340.5	F: AGTGGCGTGAACCTACAGAG<br>R: CGGTAAGTGTCGAAGCCCTC	86bp	qRT-PCR	
BAX	XM_027978592.3	F: GCCCTTTTGCTTCAGGGTTT<br>R: TCAGACACTCGCTCAGCTTC	121bp	qRT-PCR	
CASP3	XM_060406953.1	F: TCAGGGAAACCTTCACGAGC<br>R: ATCGACGGGTCCATTGGTTC	185bp	qRT-PCR	
BCL2	XM_012103831.5	F: TGGCCTTCTTTGAGTTCGGA<br>R: GGCCATACAGCTCCACAAAG	168bp	qRT-PCR	
β-actin	NM_001009784.2	F: CTTCCAGCCTTCCTTCCTGG<br>R: GCCAGGGCAGTGATCTCTTT	180bp	qRT-PCR	
LDHA	XM_042238239.1	F: ATGTTGCTGGTGTCTCCCTG<br>R: GCCCAGGATGTGTAGCCTTT	139bp	qRT-PCR	
MCT1	XM_042252647.1	F: CTGGGCATGTGGCATAATCCT<br>R: GCTGCATCGGTGACTTCTTT	177bp	qRT-PCR	
GLUT1	XM_027968628.2	F: CATTGTGGGCATGTGCTTCC<br>R: AATCTCATCGAAGGTCCGGC	142bp	qRT-PCR	
GLUT3	NM_001009770.1	F: AGGAACCCGCGGTGAAAAA<br>R: AGGCATTGGTGTCCTTCGTG	71bp	qRT-PCR	
