# Supplementary material for: Protocatechuic acid promotes lactate synthesis in Sertoli cells of Tibetan sheep through AMPK/mTOR-mediated autophagy
Source: Anim Biosci. 2026 Feb 6;39(6):250776. doi: 10.5713/ab.250776 (PMC13243928; doi:10.5713/ab.250776)
Supplement: Supplementary file 3 [file ab-250776-Supplementary-3.pdf]

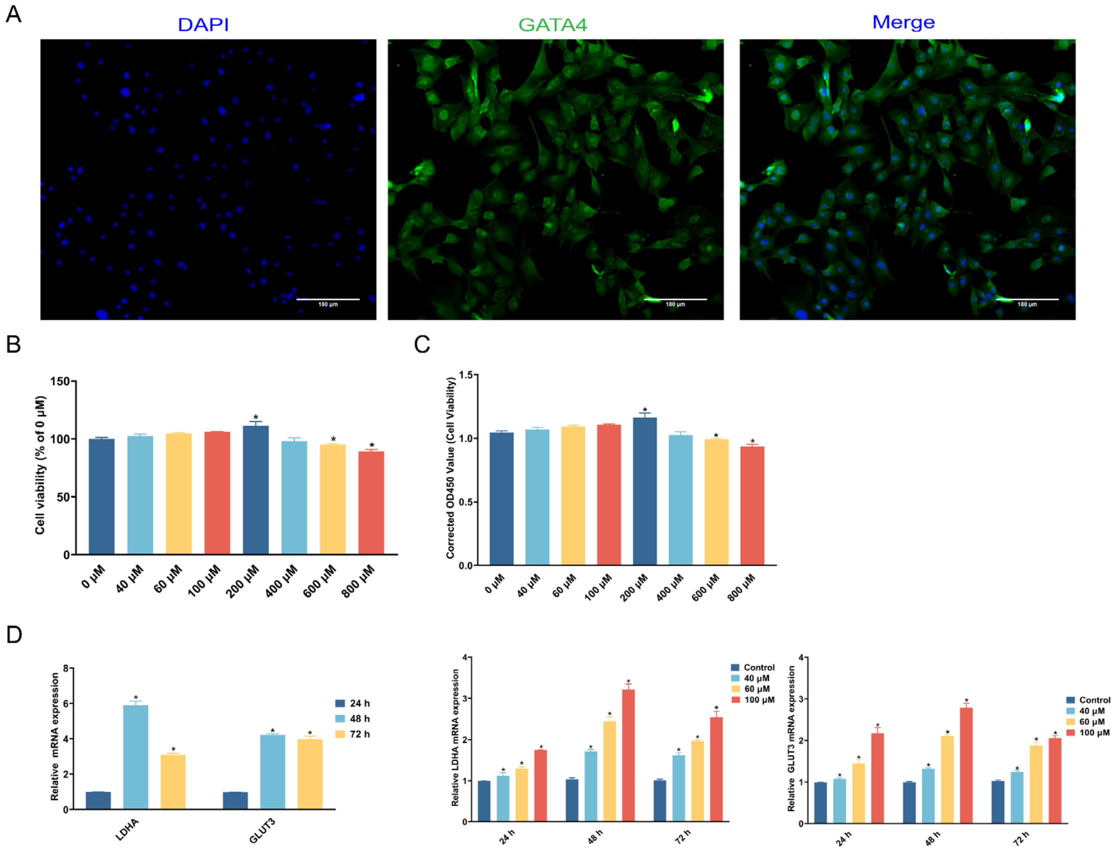

Supplement 3. Identification of Tibetan sheep primary SCs and screening of concentration and treatment duration. A: Identification of Tibetan sheep primary SCs by immunofluorescence staining for the specific marker GATA4. B: Relative cell viability (expressed as a percentage of the control group) under different PCA concentrations determined by CK-8 assay. C: Cell viability calculated from calibrated OD450 absorbance values, presented as actual readings. D: mRNA expression of *GLUT3* and *LDHA* detected by qPCR. Data are presented as the mean ± SD. **p*<0.05 vs. control group and 24 h group. SCs, Sertoli cells; PCA, protocatechuic acid; CCK-8, Cell Counting Kit-8; GLUT3, glucose transporter 3; LDHA, lactate dehydrogenase A; qPCR, quantitative real-time polymerase chain reaction; SD, standard deviation.
