# Supplementary material for: Protocatechuic acid promotes lactate synthesis in Sertoli cells of Tibetan sheep through AMPK/mTOR-mediated autophagy
Source: Anim Biosci. 2026 Feb 6;39(6):250776. doi: 10.5713/ab.250776 (PMC13243928; doi:10.5713/ab.250776)
Supplement: Supplementary file 4 [file ab-250776-Supplementary-4.pdf]

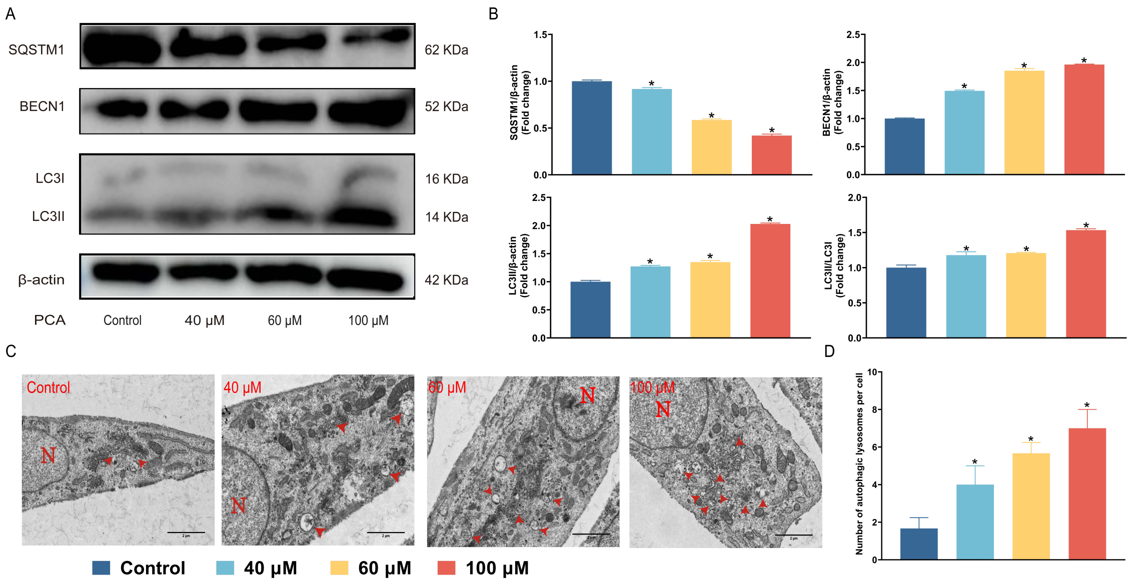

Supplement 4. PCA activates autophagy in Tibetan sheep primary SCs. A: Protein levels of SQSTM1, BECN1, and LC3II detected by Western blot. B: Quantitative analysis of protein bands in panel A. C: Autophagy levels were evaluated by transmission electron micrographs, N: nucleus. D: Quantitative analysis of panel C. Data are presented as the mean ± SD.. **p*<0.05 vs. control group. PCA, protocatechuic acid; SCs, Sertoli cells; SQSTM1, sequestosome 1; BECN1, beclin 1; LC3II, microtubule-associated protein 1 light chain 3 beta-II; SD, standard deviation.
