# Supplementary material for: Protocatechuic acid promotes lactate synthesis in Sertoli cells of Tibetan sheep through AMPK/mTOR-mediated autophagy
Source: Anim Biosci. 2026 Feb 6;39(6):250776. doi: 10.5713/ab.250776 (PMC13243928; doi:10.5713/ab.250776)
Supplement: Supplementary file 5 [file ab-250776-Supplementary-5.pdf]

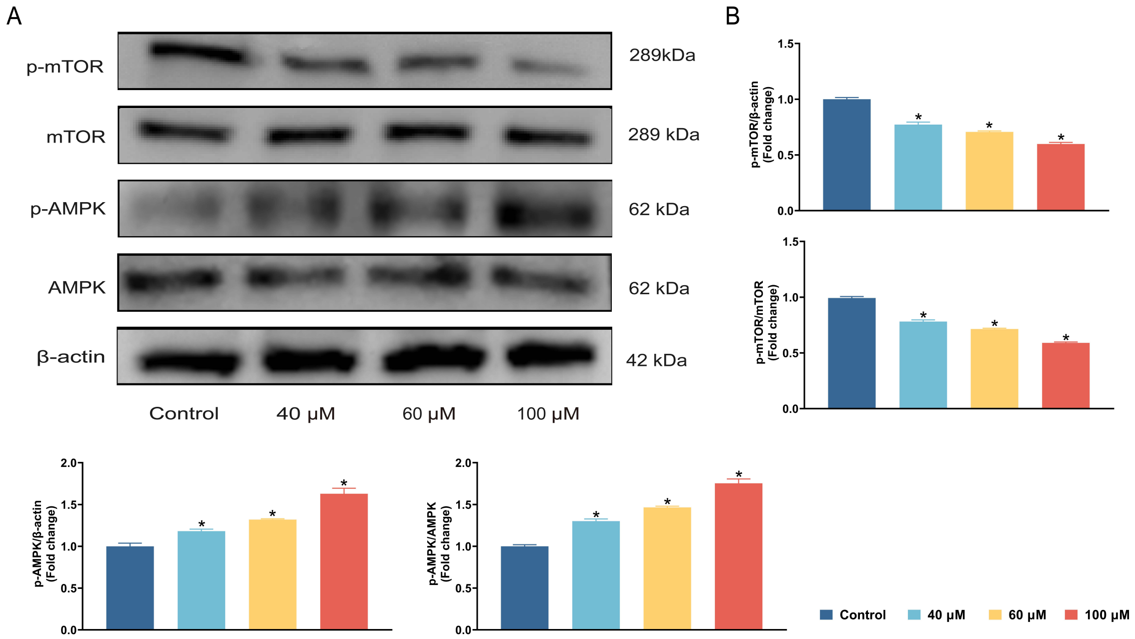

Supplement 5. PCA regulates AMPK/mTOR pathway in Tibetan sheep primary SCs.

A: Protein levels of AMPK/mTOR pathway-related p-mTOR, mTOR, p-AMPK, and AMPK detected by Western blot. B: Quantitative analysis of protein bands in panel A. Data are presented as the mean ± SD. **p*<0.05 vs. control group. PCA, protocatechuic acid; AMPK, AMP-activated protein kinase; mTOR, mechanistic target of rapamycin; SCs, Sertoli cells; p-mTOR, phosphorylated mechanistic target of rapamycin; p-AMPK, phosphorylated AMP-activated protein kinase; SD, standard deviation.
