# Supplementary material for: Protocatechuic acid promotes lactate synthesis in Sertoli cells of Tibetan sheep through AMPK/mTOR-mediated autophagy
Source: Anim Biosci. 2026 Feb 6;39(6):250776. doi: 10.5713/ab.250776 (PMC13243928; doi:10.5713/ab.250776)
Supplement: Supplementary file 6 [file ab-250776-Supplementary-6.pdf]

Supplement 6. Effects of PCA on reproductive performance of male mice

Group	Number of mated females	Number of pregnant females	Pregnancy rate (%)	Litter size (mean ± SD)
Control	20	17	85%	8.24 ± 1.09
50 mg/kg	20	17	85%	8.18 ± 0.72
100 mg/kg	20	19	95%	9.21 ± 0.85*
200 mg/kg	20	18	90%	9.06 ± 0.96*

Note: Male fertility was evaluated based on pregnancy outcomes and litter size of mated female mice. Data are presented as counts (number of mated females and pregnant females) or as mean ± SD.*p<0.05 vs. control group
