# Supplementary material for: Protocatechuic acid promotes lactate synthesis in Sertoli cells of Tibetan sheep through AMPK/mTOR-mediated autophagy
Source: Anim Biosci. 2026 Feb 6;39(6):250776. doi: 10.5713/ab.250776 (PMC13243928; doi:10.5713/ab.250776)
Supplement: Supplementary file 7 [file ab-250776-Supplementary-7.pdf]

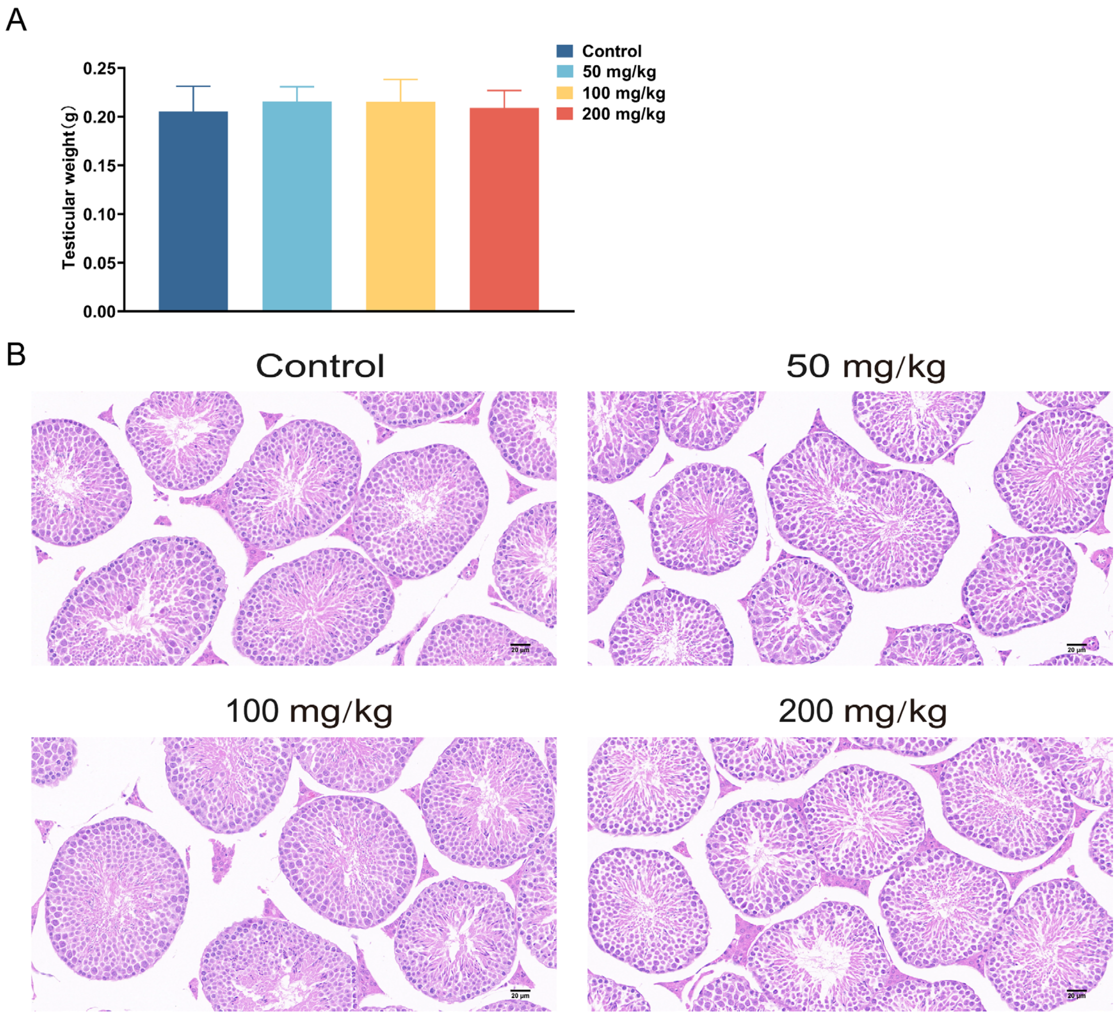

Supplement 7. Effects of PCA on testicular weight and morphology in mice. A: Testicular weight of mice. B: Testicular morphology of mice observed by HE staining. Data are presented as the mean ± SD. PCA, protocatechuic acid; HE, hematoxylin-eosin; SD, standard deviation.
