# Supplementary material for: Protocatechuic acid promotes lactate synthesis in Sertoli cells of Tibetan sheep through AMPK/mTOR-mediated autophagy
Source: Anim Biosci. 2026 Feb 6;39(6):250776. doi: 10.5713/ab.250776 (PMC13243928; doi:10.5713/ab.250776)
Supplement: Supplementary file 8 [file ab-250776-Supplementary-8.pdf]

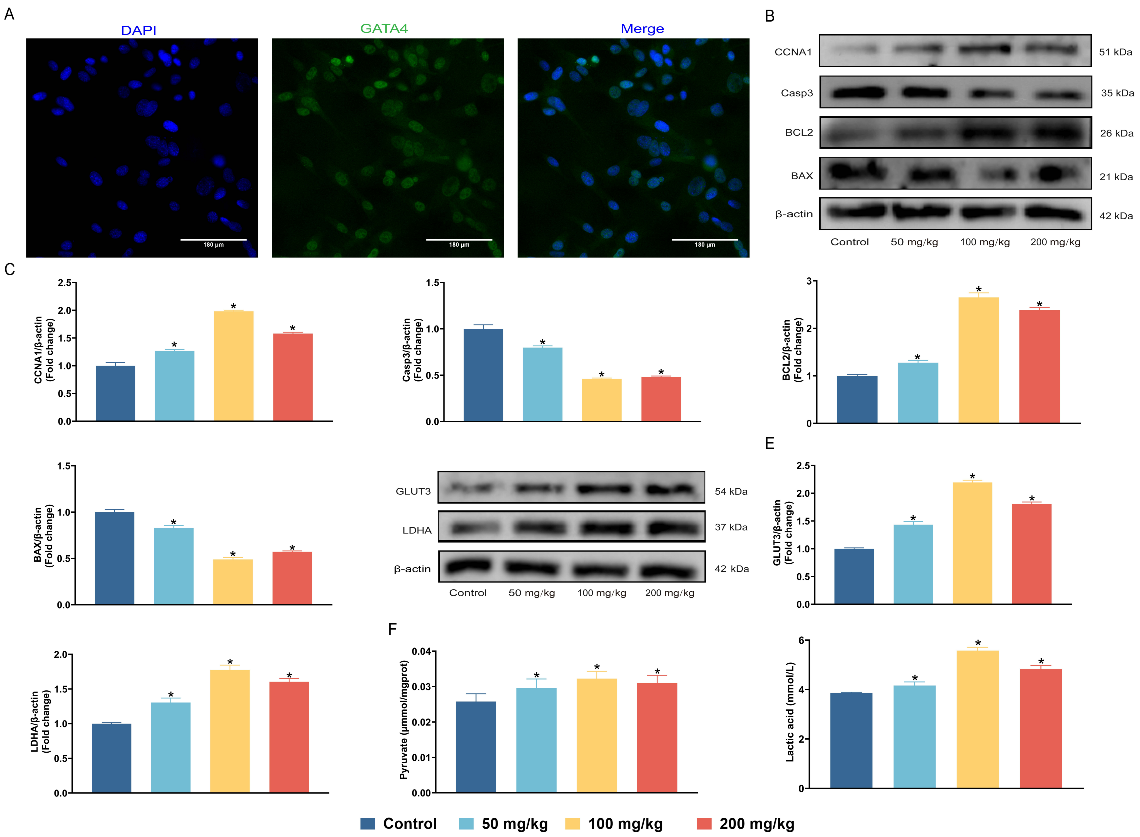

Supplement 8. PCA enhances proliferation and lactate synthesis in primary SCs of mice. A: Identification of primary SCs of mice by immunofluorescence staining for the specific marker GATA4. B: Protein levels of CCNA1, Casp3, BCL2, and BAX detected by Western blot. C: Quantitative analysis of protein bands in panel B. D: Protein levels of GLUT3 and LDHA detected by Western blot. E: Quantitative analysis of protein bands in panel D. F: Levels of pyruvate and lactate in SCs determined by commercial assay kits. Data are presented as the mean ± SD. **p*<0.05 vs. control group. PCA, protocatechuic acid; SCs, Sertoli cells; GATA4, GATA binding protein 4; CCNA1, cyclin A1; Casp3, caspase 3; BCL2, B-cell lymphoma 2; BAX, BCL2-associated X protein; GLUT3, glucose transporter 3; LDHA, lactate dehydrogenase A; SD, standard deviation.
