# Supplementary material for: Protocatechuic acid promotes lactate synthesis in Sertoli cells of Tibetan sheep through AMPK/mTOR-mediated autophagy
Source: Anim Biosci. 2026 Feb 6;39(6):250776. doi: 10.5713/ab.250776 (PMC13243928; doi:10.5713/ab.250776)
Supplement: Supplementary file 9 [file ab-250776-Supplementary-9.pdf]

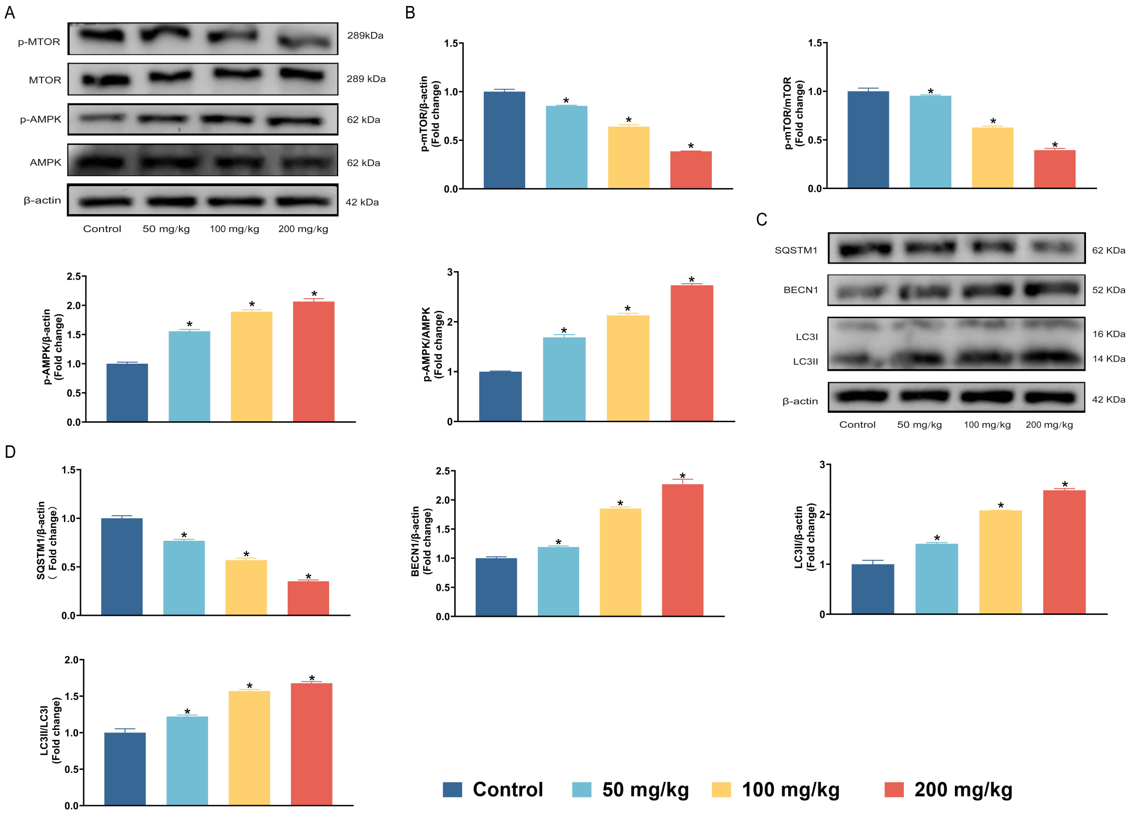

Supplement 9. PCA regulates AMPK/mTOR signaling pathway and enhances autophagy in primary SCs of mice. A: Protein levels of p-mTOR, mTOR, p-AMPK, and AMPK detected by Western blot. B: Quantitative analysis of protein bands in panel A. C: Protein levels of SQSTM1, BECN1 and LC3II detected by Western blot. D: Quantitative analysis of protein bands in panel C. Data are presented as the mean ± SD. *p<0.05 vs. control group. PCA, protocatechuic acid; AMPK, AMP-activated protein kinase; mTOR, mechanistic target of rapamycin; SCs, Sertoli cells; p-mTOR, phosphorylated mechanistic target of rapamycin; p-AMPK, phosphorylated AMP-activated protein kinase; SQSTM1, sequestosome 1; BECN1, beclin 1; LC3II, microtubule-associated protein 1 light chain 3 beta-II; SD, standard deviation.
